# Integrated enhanced recovery after surgery protocol in radical cystectomy for bladder tumour—A retroprospective study

**DOI:** 10.1002/bco2.438

**Published:** 2024-10-06

**Authors:** Waseem Ashraf, Arif Hamid, Sajad Ahmad Malik, Rouf Khawaja, Sajad Ahmad Para, Mohammad Saleem Wani, Saqib Mehdi

**Affiliations:** ^1^ MCh Scholar Department of Urology and Renal Transplant Sher‐i‐Kashmir Institute of Medical Sciences Srinagar Kashmir India

**Keywords:** bladder tumour, ERAS, radical cystectomy

## Abstract

**Introduction:**

Enhanced recovery after surgery (ERAS) is a patient‐centerd, evidence‐based approach to improve postoperative outcomes. The protocol involves multidisciplinary collaboration and standardisation of perioperative interventions. ERAS has shown positive results in reducing hospitalisation and complications.

**Methods:**

The study conducted in the Department of Urology was a retro‐prospective study. It included an ERAS cohort group of 47 patients, studied prospectively from May 2021 to May 2023. These patients were compared to a historical cohort of 47 consecutive patients who underwent radical cystectomy with traditional care before the ERAS pathway was implemented. The primary outcome was hospital length of stay (LOS). Secondary outcomes included perioperative management, time to recovery milestones and complications.

**Results:**

Implementation of ERAS pathway for radical cystectomy was associated with reduced hospital LOS (mean LOS 16.19 ± 2.53 days vs. 10.26 ± 3.33 days 7 days; *p* < 0.0001), reduced time to key recovery milestones, including days to first flatus (3.17 vs. 2.68; *p* = 0.013) and days to first solid food (5.19 vs. 3.45 *p* value < 0.0001), first stool (5.53 vs. 4.23; *p* < 0.0001), reductions in some complications like postoperative ileus (*p* value = 0.021) and need for total parental nutrition (*p* value = 0.023).

**Conclusion:**

In conclusion, the implementation of the integrated approach facilitates a more efficient recovery process, potentially reducing healthcare costs and enhancing patient comfort.

## 1 INTRODUCTION

Enhanced recovery after surgery (ERAS) (formerly ‘fast track surgery’) was first introduced in 1997 by Professor Henrik Kehlet, Copenhagen University Hospital in colorectal surgery as part of a multimodal approach to postoperative recovery.^1^, ^2^, ^3^ Kehlet suggested that ‘while no single technique or drug regimen has been shown to eliminate postoperative morbidity and mortality, multimodal interventions may lead to a major reduction’ and went on to suggest pre‐, intra‐ and postoperative surgical risk factors that may be addressed through coordinated perioperative protocols. He started making inquiries about whether we offered the surgical patients the best treatment and care, and if so, whether was it evidence‐based? These considerations involved the whole pathway from identification of a need for surgery, preparation for surgery, perioperative procedures and postoperative care. Following this, Kehlet published a trial that reported a mean postoperative hospital stay of 48 h after elective sigmoid resection.^4^ Though this study included just 16 patients, fast‐track surgery reduced post‐surgical hospitalisation by 3–8 days.^4^ The concept was that the key pathogenic factor in postoperative morbidity is the surgical stress response with subsequent increased demands on organ function. These changes in organ function were believed to be mediated by trauma‐induced endocrine metabolic changes and activation of several biological cascade systems, the surgical stress response.^5^ So, in order to understand postoperative morbidity, it was necessary to understand the pathophysiological role of the various components of the surgical stress response and clarify if modification of such responses and other risk factors could improve surgical outcomes.^5^ This was the onset of three decades of extensive research across surgical specialties to clarify whether a multi‐professional approach and multimodal procedure‐specific interventions may significantly reduce the undesirable sequelae of surgical injury, improve recovery and have a profound reduction in postoperative morbidity and overall costs. In 2001 came the formation of the ERAS Study Group—a group of six surgeons assembled by Professors Ken Fearon and Ollie Ljungqvist and including Professor Kehlet, which aimed to produce and interpret the best available evidence to fine‐tune fast track surgery.^6^, ^7^

In 2005, the ERAS study group published an evidence‐based ERAS protocol for colorectal cancer patients.^8^ ERAS refers to surgery‐specific pathways that are patient‐centred and multidisciplinary and that aim to standardise and integrate a range of perioperative interventions throughout the perioperative period while incorporating the best available evidence‐based medicine. ERAS pathways seek to attenuate the surgical stress response, optimise physiologic and organ function and achieve early recovery.^9^, ^10^, ^11^ Things that can prevent hospital discharge after surgery are the need for parenteral analgesia, gut dysfunction with consequent and perhaps contributory intravenous fluid administration and lack of mobility. These factors often overlap and interact to delay recovery and discharge from the hospital. The fundamental elements of an ERAS pathway are designed to target these issues and address the pre‐, intra‐ and postoperative phases of the patient's journey.^12^

Bladder cancers are the second most common of the genitourinary system.^13^ Approximately 20–40% of them are muscle invasive at the time of diagnosis.^14^ The primary goal of bladder cancer treatment is to minimise mortality and morbidity while obtaining the best oncological results. Fortunately, morbidity and mortality from Radical cystectomy have decreased in recent years due to advances in surgical technique, anaesthesia and postoperative procedures.

Although various methods are used for urinary diversion, the ileal conduit is currently the most preferred method. Early complications such as nausea, vomiting, fever and ileus have been reported in patients after the ileal conduit procedure. These complications affect the length of hospital stay and increase the cost of the operation.^15^

In urology, the role of ERAS was evaluated in 2013, and it was concluded that ERAS had not yet been widely implemented in urology and that evidence for individual interventions was limited or unavailable.^16^ The core question ‘why is the patient still in the hospital?’ became the driving force to optimise the surgical pathways.^17^ The initial guidelines released by the ERAS society in 2013 outlined 22 targetable items, of which only seven items had sufficient evidence. These included oral mechanical bowel preparation, minimally invasive approach, perioperative fluid management, nasogastric intubation, urinary drainage, prevention of postoperative ileus and prevention of postoperative nausea/vomiting. In the colorectal field, meta‐analyses demonstrated high‐level evidence for reduced complication rates and postoperative hospital length of stay (LOS) associated with ERAS pathways. The concepts, however, rested on the same five basic components: patient information, goal‐directed fluid therapy, nutrition, pain management and early mobilisation and carefully described by the ERAS group, and evidence was collected and monitored from different surgical specialities and populations.^18^ The concept clearly encouraged a multi‐modal and multi‐professional approach to surgery and surgical care, and continuous nursing involvement, especially inpatient information, nutritional care and early mobilisation, is of utmost importance.^19^, ^20^, ^21^ The improved understanding of the pathophysiology of postoperative recovery within an integrated multi‐professional and multi‐modal approach almost immediately resulted in positive results across surgical procedures with a reduction in hospitalisation and medical complications (in some specialities) and without increased re‐admission rates. Recently, new chapters of the ERAS Society have been launched around the world, and importantly, a special section of nursing care has been formed to promote education and provide support to ERAS coordinators, which most often are dedicated nurses in the surgical field.^21^ Nevertheless, adoption of ERAS in the urologic community was slow, and many criticised the lack of prospective randomised controlled trials (RCTs) evidence in this space.

Nevertheless, adoption of ERAS in the urologic community was slow, and many criticised the lack of prospective RCTs evidence in this space. In the past decade, various institutions have adopted modified versions of the ERAS protocol that was introduced in 2013.^22^ Since then, increased interest in the ERAS pathway has resulted in studies that provide cystectomy‐specific evidence for the use of ERAS on optimising perioperative care.^23^ In 2018, the American Urological Association (AUA) published comprehensive guidelines for optimising postoperative outcomes in urologic surgery, which was based on the ERAS movement.^24^ Nevertheless, ERAS remains a growing movement for which standardisation has not yet been achieved.^25^

## 2 MATERIALS AND METHODS

The present study is a retro‐prospective study. It was a single‐centre study conducted at Sher‐I‐Kashmir Institute of Medical Sciences (SKIMS), Soura Srinagar, in the department of urology (SKIMS). The study on ERAS cohort group of patients was conducted prospectively from May 2021 to May 2023, and a total of 47 patients were acquired and compared with a historical cohort of 47 consecutive patients undergoing radical cystectomy with traditional care prior to the implementation of the ERAS pathway.

Inclusion criteria:

- Patients undergoing radical cystectomy with urinary diversion/bladder reconstruction for bladder cancer were included in the study.

Exclusion criteria:

- The inclusion criteria were not met.
- Patients who underwent cystectomy for reason other than for bladder cancer.
- Patients who under radical cystectomy with cutaneous urinary diversion.
- Patients not giving consent.
- Patients lost to follow up.

Primary outcome:

- The primary outcome of this study is hospital LOS after surgery, defined as the number of postoperative nights in the hospital.

Secondary outcome:

- The secondary outcome of this study was time to first bowel movement, time to ingestion of liquid/solid food, estimated blood loss, postoperative opioid requirements, complications and readmissions.

### 2.1 Statistical analysis

Statistical analysis will be done by using SPSS V24. All categorical variables will be observed by frequency and percentage. Also, further statistical analysis will be done by knowing the normality of distribution and then apply the parametric and nonparametric test (*t* test, chi square test, analysis of variance [ANOVA]). All results will be discussed at 5% level of significance, that is, *p* < 0.05.

Sample size estimation:

With estimation and proportion in finite population, taking

Alpha(a) = 0.05,

Estimated proportion(p) = 0.05,

Estimated error(d) = 0.04,

Sample size = 30,

Having 90% power of study.

### 2.2 Traditional care

Patients in the traditional care group were cared for according to provider preference as there was no standardised pathway between surgeons or anesthesiologists before initiation of ERAS. For example, epidural analgesia was common but not universal or standardised. Multimodal analgesics were given at provider discretion and were not common. The pre‐ and postoperative elements of the enhanced recovery pathway were not in place. Many of the potential advantages related to the intraoperative management may not have been fully realised if patients were not prepared preoperatively and encouraged to ambulate and eat early postoperatively.

### 2.3 The integrated ERAS pathway

This pathway is now the standard of care for all patients undergoing radical cystectomy at SKIMS Soura Department of Urology. The pathway features preoperative education, multimodal analgesia, thoracic epidural, optimal fluid management and early mobilisation and PO intake after surgery. The ERAS protocol includes preoperative, intraoperative and postoperative components. The summary of comparison between the two groups is shown in Table 1.

**TABLE 1:** Summary of ERAS programme versus traditional care.

Variables	Integrated ERAS	Traditional care
Preoperative		
1. Counselling and education:	Patient educated about the pathway in the surgical clinic and counselled about the stoma with stoma site marked by a stoma therapist	Advice and counselling was given by the operating surgeon
2. Bowel preparation:	Preoperative bowel preparation is not routinely used however patient has to be given enema night before surgery	Oral mechanical bowel preparation (MBP) using polyethylene glycol was routinely given
3. Minimise prolonged fasting:	Patients allowed clear fluids until 4 h before the start of surgery and solids 6 h before surgery. 500 mL (100 g) carbohydrate drink 6 h before surgery (clear fast)	Patients were kept on an overnight fast and clear liquids for 48 h before surgery.
4. Preoperative oral adjunctive analgesics:	Gabapentin 300 mg night before surgery	No preoperative analgesic was used
5. Incentive spirometry:	For chest physiotherapy 1 day before surgery to be continued post operatively	No lung exercise was used preoperatively.
Intraoperative		
1. Multimodal opioid sparing analgesia:	No IV opioids after induction without discussion with attending anaesthesiologist	IV opioids after induction were routinely used
2. Short acting analgesia	Yes	No
3. Regional anaesthesia –	Combined spinal epidural anaesthesia was used.	General anaesthesia was preferred
4. Blood products transfused as needed.	Yes	Not preferred
5. Drains and NG tubes	Limit and minimise use of drains, catheters and NG tubes	Drains and NG tubes were routinely used
6. Temperature management/normothermia:	Intraoperative use of temperature management units (bair hugger/bair paws) for prevention of hypothermia and its consequences	No such intraoperative temperature regulating devices were used.
7. Smaller incisions/extraperitoneal radical cystectomy to minimise gut handling and postoperative ileus	Extraperitoneal radical cystectomy was done in most cases	Intraperitoneal approach was used in most cases of radical cystectomy
Postoperative		
1. Early oral diet:	Patients encouraged to drink liquids in the evening. In order to prevent ileus chewing gum and potassium rich fluids recommended after surgery. IV fluids discontinued once adequate oral intake is achieved, usually the first morning after surgery	No such protocol was used
2. Epidural analgesia:	Patients transitioned to oral analgesics after removal of epidural catheter usually after 72 h after surgery.	Epidural analgesia was not used in the postoperative period
3. Early mobilisation:	Encouraged to be out of bed on the day after surgery and for at least 6 h on every subsequent day. Pt is instructed to stay seated 60% of the time	Patients were mobilised late in the postoperative period
4. Early stent removal:	Stents are to be avoided or removed early on 3rd POD	Stents were removed late from 10th to 14th postoperative period
5. Chest physiotherapy/incentive spirometry:	Incentive spirometry to be continued postoperatively	No
6. Thromboembolic prophylaxis:	Compression stockings and thromboembolic prophylaxis to be started on POD1 and continued to complete ambulation.	Prophylaxis for thromboembolism was used only till 1 month of postoperative period.

Abbreviation: ERAS, enhanced recovery after surgery.

## 3 RESULTS

The study entitled ‘Integrated enhanced recovery after surgery (eras) protocol in radical cystectomy for bladder tumor‐a retroprospective study’ was conducted in the department of urology and Kidney Transplant, Sher‐i‐Kashmir Institute of Medical Sciences, Soura, Srinagar. The study on ERAS cohort group of patients was conducted prospectively from May 2021 to May 2023, and a total of 47 patients were enrolled in this study in the integrated ERAS group and were be compared with a historical cohort of 47 consecutive patients undergoing radical cystectomy with traditional care prior to the implementation of the ERAS pathway.

The comparison of baseline characteristics between the two groups is shown in Table 2. Of the total 94 patients included in the study the age group of maximum number of patients undergoing radical cystectomy for bladder tumor was 60–79 years in both the case and control groups. Mean age ± standard deviation of the patients in the ERA cohort was 60.04 ± 12.67 years, whereas in the traditional cohort, it was 60.36 ± 13.26 years with no statistical significance (*p* value = 0.905). Majority of the patients in both the integrated ERAS (91.49%, *n* = 43) and traditional group (89.36%, *n* = 42) were males. In the Integrated ERAs group, there were 43 male patients and 4 female patients. In the traditional care group, there were 42 male patients and 5 female patients with statistically insignificant comparison between the two groups (*p* value = 1). In the Integrated ERAs group, majority of the patients were underweight (53.19%, *n* = 25), whereas in the traditional group, majority of the patients were of normal weight (53.19%, *n* = 25) with no statistical significance between the two groups (*p* value = 0.463). Comparing the two groups, it appears that a slightly higher proportion of patients in the traditional care had extravesical disease (or ≥pN1) ≥ pT3 (68.1%) compared with the integrated ERAs group (61.7%), and majority of the patients in the integrated ERAS group had organ confined disease (31.9%) compared with the traditional care (27.66%) with no statistical significance between the two groups (*p* value = 0.782). Transitional cell carcinoma is the most common type of urothelial malignancy in both the comparison groups (81% vs. 87.23%), while mixed/variant histology is the second most common type in both the groups (12.8% vs. 6.38%) with insignificant difference between the two groups (*p* value = 0.619). The comorbidity status of both the groups are comparable with hypertension being the most common comorbidity in both the two groups (36.2% vs. 42.6, *p* value = 0.528); 36.2% patients in the integrated ERAS group and 40.4% patients in the traditional group had no comorbidity. Based on the *p* value, there is no statistically significant difference in the prevalence of these comorbidities between the two groups. Majority of the patients in both the ERAS group (59.57%, *n* = 28) and the traditional care (55.32%, *n* = 26) belonged to ASA score 2 with no statistical significance between the two groups (*p* value = 0.718), and radical cystectomy with ileal conduit was most commonly done diversion procedure in both the two cohort groups.

**TABLE 2:** Comparison of demographic profile between the two groups.

	Integrated ERAS	Traditional care	*p* value
Age group (mean ± SD)	60.04 ± 12.67	60.36 ± 13.26	0.905
Sex			
Male	43	42	1.0000
Female	4	5	
Body mass index			
Underweight <18.5	25	21	
Norm weight 18.5–24.9	22	25	0.463
Overweight >25	0	1	
Staging, *n* (%)			
Organ‐confined disease (pN0) ≤ pT2,	15	13	
Extravesical disease (or ≥pN1) ≥ pT3	29	32	0.782
Metastatic disease (m)	3	2	
Pathological finding			
Transitional cell carcinoma	38	41	
Adenocarcinoma	2	1	0.6192
Squamous carcinoma	1	2	
Variant histology/mixed	6	3	
Comorbidities			
Hypertension	17	20	0.528
Diabetes mellitus	6	7	0.766
Renal insufficiency	3	2	0.647
Respiratory comorbidity	11	10	0.806
Cardiovascular disease	8	7	0.780
No comorbidity	17	19	0.672
ASA score, *n* (%)			
ASA‐1	1	0	
ASA‐2	28	26	
ASA‐3	10	11	0.718
ASA‐4	8	10	
Type of diversion, *n* (%)			
Radical cystectomy with Ileal conduit	44	45	1
Radical cystectomy with orthotopic neobladder	3	2	

Abbreviation: ERAS, enhanced recovery after surgery.

From Table 3, the mean ± SD for drain removal in the integrated ERAS was 3.53 ± 0.78 days. While in the traditional group, mean ± SD for drain removal is 11.98 ± 2.38 days. The *p* value is <0.0001, indicating a highly significant difference between the two groups regarding drain removal.

**TABLE 3:** Postoperative milestones.

Postoperative milestones	Integrated ERAS (*n* = 47)	Traditional care (*n* = 47)	*p* value
Mean ± SD drain removal	3.53 ± 0.78	11.98 ± 2.38	<0.0001
Mean ± SD stent removal	5 ± 0.96	12.70 ± 1.99	<0.0001
Mean ± SD length of hospital stay	10.26 ± 3.33	16.19 ± 2.52	<0.0001
Median ± SD length of hospital stay	10 ± 3.33	17 ± 2.53	<0.0001

Abbreviation: ERAS, enhanced recovery after surgery.

Similarly, the mean ± SD for stent removal in the integrated ERAS was 5 ± 0.96 days. While in the traditional group, mean ± SD for stent removal was 12.7 ± 1.99 days. The *p* value is <0.0001, indicating a highly significant difference between the two groups (Table 3).

**FIGURE 1:**
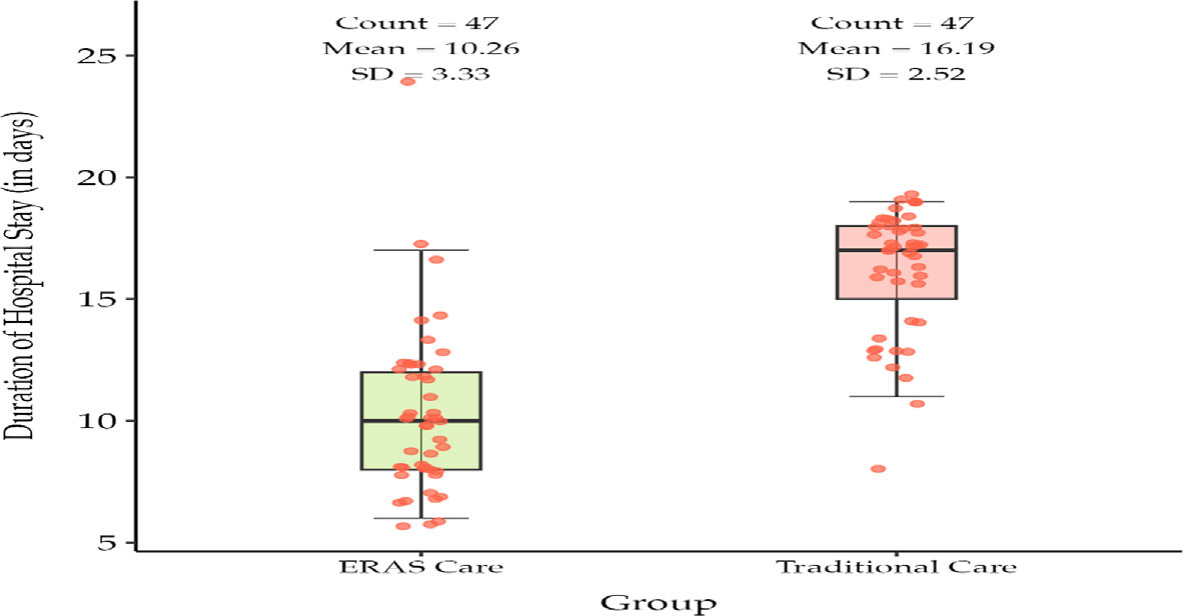
Box and whisker plot comparing postoperative length of hospital stay (in days).

In the Integrated ERAs group, the mean ± SD length of hospital stay was 10.26 ± 3.33 days, whereas the median ± SD length of hospital stay was 10 ± 3.33 days. In the traditional care group, mean ± SD length of hospital stay was 16.19 ± 2.53 days, whereas the median ± SD length of hospital stay was 17 ± 2.53 days (Figure 1). The *p* values for both the mean and median comparisons are <0.0001, indicating a highly significant difference in the length of hospital stay between the two groups as shown in Table 3.

**FIGURE 2:**
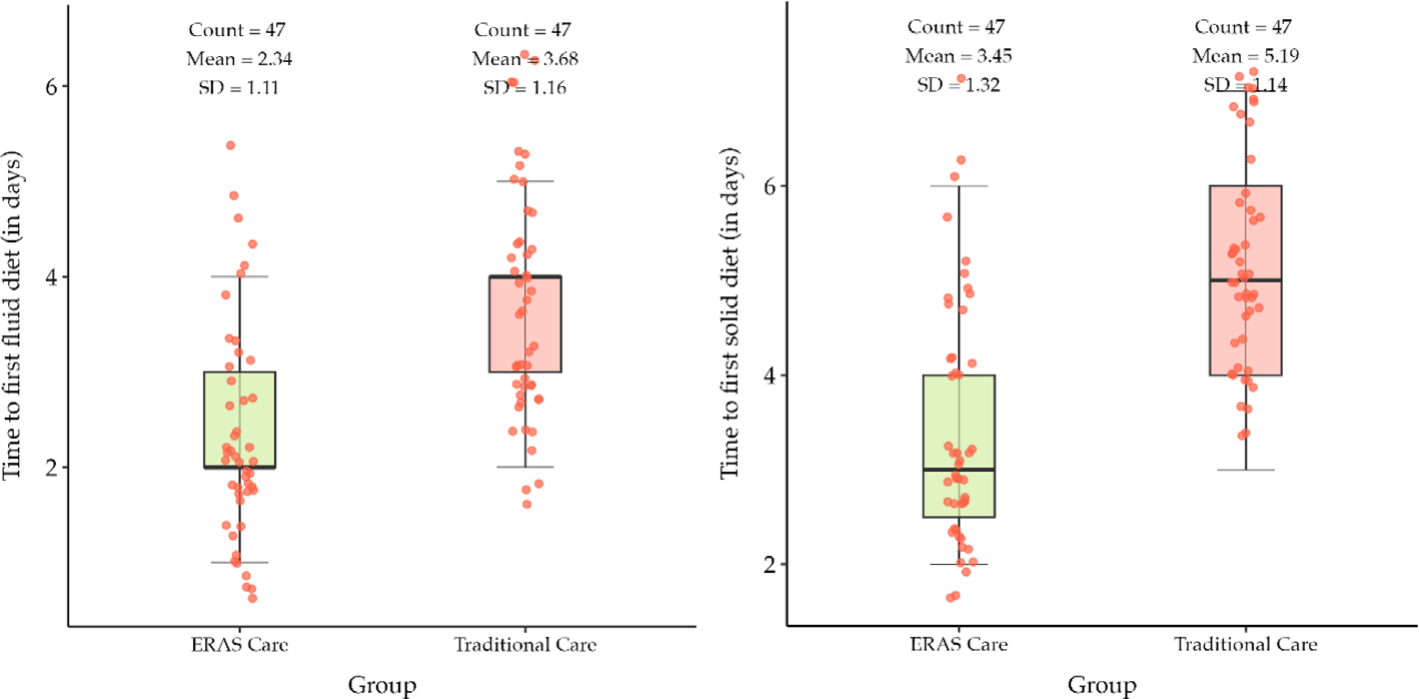
Box and whisker plot postoperative functional recovery (in days).

**FIGURE 3:**
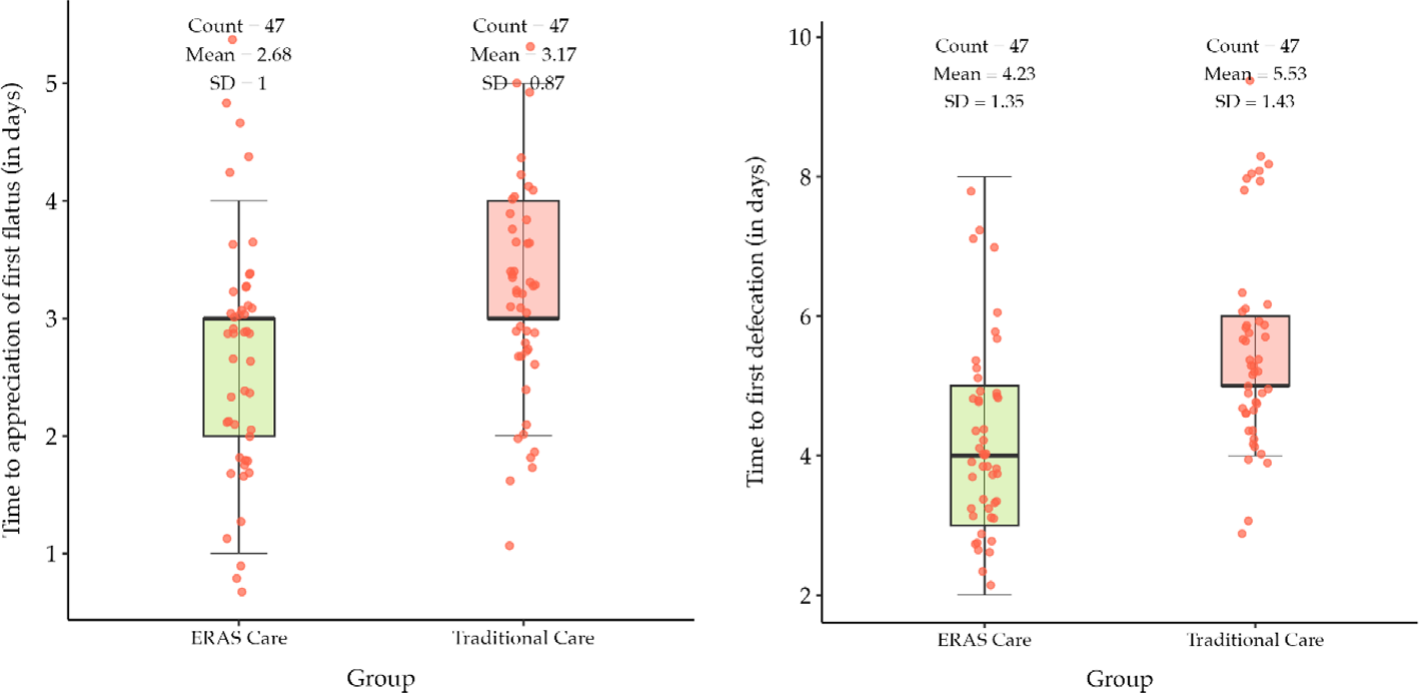
Box and whisker chart comparing postoperative bowel recovery (in days).

In Table 4, the integrated ERAs group mean day ± SD to first fluid diet was 2.34 ± 1.11, whereas in the traditional care group, mean day ± SD to first fluid diet was 3.68 ± 1.16. Similarly in the integrated ERAs group, mean day ± SD to first solid diet was 3.45 ± 1.32, while in the traditional care group, mean day ± SD to first solid diet was 5.19 ± 1.14 (Figure 2). The *p* value provided is <0.0001, in both the groups indicating a highly significant difference in the timing to functional recovery. In the integrated ERAS cohort, the mean ± SD to first flatulence was 2.68 ± 1, whereas in the traditional care, it was 3.17 ± 0.87 (*p* value = 0.013). The mean ± SD to first defecation in the integrated ERAS was 4.23 ± 1.35, whereas in the traditional care, it was 5.53 ± 1.42 (*p* value < 0.0001) showing a statistically significant postoperative bowel recovery (Figure 3).

**TABLE 4:** Postoperative functional and bowel recovery.

Mean day ± SD functional recovery	Integrated ERAS (*n* = 47)	Traditional care (*n* = 47)	*p* value
First fluid diet	2.34 ± 1.11	3.68 ± 1.16	<0.0001
First solid diet	3.45 ± 1.32	5.19 ± 1.14	<0.0001
First flatulence	2.68 ± 1	3.17 ± 0.87	0.013
First defecation	4.23 ± 1.35	5.53 ± 1.42	<0.0001

Abbreviation: ERAS, enhanced recovery after surgery.

Table 5 shows the complication rate between the two groups in the integrated ERAs cohort compared with the traditional cohort showing a statistically significant difference in postoperative ileus (31.91% vs. 55.32%, *p* value = 0.021) and need for TPN (36.3% vs. 55.32, *p* value = 0.023). Rest of the complications are statistically insignificant (*p* value > 0.05). There was statistically insignificant reduction in postoperative fever (17.02% vs. 21.28, *p* value = 0.60), surgical site infections (10.64 vs. 14.9, *p* value = 0.536), intraabdominal collection (6.38% vs. 4.26, *p* value = 0.645), SSI requiring drainage (4.26 vs. 2.13, *p* value = 0.557), respiratory failure requiring ventilatory support (10.64 vs. 14.9, *p* value = 0.536) and multiorgan failure (4.26% vs. 6.38%, *p* value = 0.645).

**TABLE 5:** Postoperative complications.

Clavien Dindo grade	Postoperative complications	Integrated ERAS	Traditional care	*p* value
1	Postoperative ileus managed conservatively	15	26	0.021
	Fever managed with routine antibiotics	8	10	0.600
2	Requiring blood transfusion	5	6	0.748
	Surgical site infection not requiring drainage	5	7	0.536
3	Need for total parental nutrition	17	28	0.023
	Collection treated radiologically	3	2	0.645
	SSI requiring drainage	2	1	0.557
4	Respiratory failure requiring ventilator support	5	7	0.536
	Multiorgan failure	2	3	0.645
5	Death	3	4	0.694

Abbreviation: ERAS, enhanced recovery after surgery.

Table 6 shows the 30 day mortality rate of ERAS cohort compared with traditional cohort with no statistical significance (6.38% vs. 8.51%, *p* value = 1).

**TABLE 6:** Postoperative mortality.

	Integrated ERAS (47)	Traditional care (47)	*p* value
Dead	3	4	1
Alive	45	44	
Mortality rate	6.38%	8.51%	

Abbreviation: ERAS, enhanced recovery after surgery.

## 4 DISCUSSION

The study entitled ‘Integrated enhanced recovery after surgery (ERAS) protocol in radical cystectomy for bladder tumor‐a retroprospective study’ was conducted in the Department of urology and Kidney Transplant, Sher‐i‐Kashmir Institute of Medical Sciences, Soura, Srinagar. A total of 94 patients of carcinoma bladder, who were admitted in the department, were enrolled for this study. The study on ERAS cohort group of patients was conducted prospectively from May 2021 to May 2023, and a total of 47 patients were enrolled in this study in the integrated ERAS group and were compared with a historical cohort of 47 consecutive patients undergoing radical cystectomy with traditional care prior to the implementation of the ERAS pathway.

In our study, most patients in both the Integrated ERAS and traditional care groups have similar age distributions. The majority of individuals in both groups fall within the 60–79 age range. In the integrated ERAs group, the mean ± SD age was 60.04 ± 12.67 years, whereas in the traditional group, it was 60.36 ± 13.26 years. The *p* value is 0.905, which suggests that there is no significant difference in the mean age between the Integrated ERAs and Traditional care groups. Nayak and colleagues^26^ in 5‐year study on contemporary outcomes of open radical cystectomy reported that the mean age of patients undergoing radical cystectomy with diversion was 57.3 ± 10.9 in the Indian population. These results suggest that our study population is representative of the general patient population undergoing this procedure.

Majority of the patients of carcinoma bladder in both the integrated ERAS (91.49%, *n* = 43) and traditional group (89.36%, *n* = 42) were males. This gender predominance is because males are disproportionately affected by the disease. In our study, we found that carcinoma bladder was nine times more common in men than in women. This male predominance in bladder cancer is similar to the other studies done in the Indian population. Gupta and colleagues^27^ in their study on the impact of age and gender on the clinicopathological characteristics of bladder cancer in the Indian population found that the male‐to‐female ratio was 8.6:1, which is slightly less than that reported by us, and in the world literature, the reported male to female ratio is 3:1–5:1 (Siegel and colleagues).^28^

Patients in our study were categorised preoperatively on the basis of body mass index (BMI) to access their nutritional status prior to radical cystectomy. Most of the patients undergoing radical cystectomy with ideal conduit were either underweight (BMI < 18.5) or normal weight (BMI = 18.5–24.9). In the Integrated ERAs group, the majority of the patients were underweight (53.19%, *n* = 25), whereas in the traditional group, majority of the patients were of normal weight (53.19%, *n* = 25) with no statistical significance between the two groups (*p* value = 0.463).

In our study, most of the patients undergoing radical cystectomy with ileal conduit for bladder cancer had extravesical disease (≥pT2, ≥N1) at histopathology. This is in concordance with the study done by Hendri et al.^29^ in which he found that the majority of the patient undergoing radical cystectomy had extravesical disease at final histopathology.

Transitional cell carcinoma was the most common type of urothelial malignancy in both groups (81% vs. 87.23%), while non‐urothelial including mixed/variant histology constituted 19% in the ERAS Cohort and 12.7% in the traditional cohort. This is in accordance with the previous studies suggesting transitional cell carcinoma being the predominant histological subtype of bladder cancer accounting for about 85%–90% of cases of the bladder.^30^, ^31^ Non‐urothelial malignancy including variant histology constituted 10–25% of all bladder malignancy in most studies.^32^, ^33^

In our study, majority of the patients had some comorbidity. Cancer patients often have comorbidities that may impact treatment decisionmaking, prognosis and survival outcomes. The comorbidity severity strongly influences survival in a dose‐dependent fashion independent of the cancer stage. Bladder cancer patients present with significant competing risks, as patients are often elderly and/or have coexisting diseases that impact morbidity and mortality. The study of comorbid status in carcinoma bladder and its impact on prognosis was done in several studies by Piccirillo et al.,^34^ William et al.,^35^ Eisenberg et al.^36^ and Noon et al.^37^

Patients undergoing radical cystectomy were categorised preoperatively on admission before surgery on the basis American Society of Anaesthesiologists (ASA) physical status classification system. The majority of the patients in both the ERAS group (59.57%, *n* = 28) and the traditional care (55.32%, *n* = 26) belonged to ASA score 2 (patients with mild systemic disease) followed by ASA score 3 (patients with severe systemic disease). Similar findings were noted in the study conducted by Hendri et al.^29^ in the comparison of ERAS care with traditional care observing that the majority of the carcinoma bladder patients undergoing radical cystectomy had mild systemic disease (ASA score 2).

In our study, the most common diversion method in radical cystectomy was the ileal conduit followed by the orthotopic neobladder (ONB). In a review article on urinary diversion after radical cystectomy for bladder cancer, Lee et al.^38^ concluded that Ileal conduits represent the fastest, easiest, least complication‐prone and most commonly performed urinary diversion globally in concordance with our practice.

The mean ± SD for drain removal in the integrated ERAS was 3.53 ± 0.78 days. While in the traditional group, mean ± SD for drain removal is 11.98 ± 2.38 days. The *p* value is <0.0001, indicating a highly significant difference between the two groups regarding drain removal. Hanna et al.^39^ in a retrospective cohort study on 296 patients (146 non‐ERAS patients vs. 150 ERAS patients) undergoing radical cystectomy and urinary diversion did the early drain removal as a part of ERAS cohort resulting in a significant decrease in length of hospital stay. Similar results on the impact of early drain removal in radical cystectomy in ERAS were obtained by Moschini et al.,^40^ Özdemir et al.^41^ and Sung and Yuk.^42^

Similarly, the mean ± SD for stent removal at ureteroenteric anastomosis in the integrated ERAS is 5 ± 0.96 days as compared to 12 ± 1.99 days in the traditional group (*p* value < 0.0001). Beano et al.^43^ conducted a study on the safety of decreasing ureteral stent duration following radical cystectomy is a safe, non‐inferior and more practical stent care option. In their study, they decreased the median stent duration from 15.5 to 5 days in conformity with our protocol of stent removal. The author concluded that shortening stenting duration was linked to decreased total 90‐day readmissions and total UTIs without increased ureteroenteric stricture rates. Donat et al.^44^ concluded in their study that stent use is associated with a significantly higher incidence of infectious complications and significantly increased odds for 30‐day ureteroanastomosis associated complications.

In our study, the median LOS decreased from 17 days in the traditional cohort of patients to 10 days in the integrated ERAS group (*p* value < 0.0001) to the benefit of the healthcare system in terms of cost and resource utilisation. Semerjian et al.^45^ in their study on Hospital Charges and LOS Following Radical Cystectomy in the Enhanced Recovery concluded that implementation of the ERAS pathway resulted in significantly reduced LOS and total hospital charge, with comparable rates of complication and readmission, highlighting the need for ERAS pathways in patients undergoing Radical cystectomy. This decrease in the length of hospital stay on implementation of ERAS protocol was also seen in studies by Pramod et al.^46^ and Wessels et al.^47^

There were also significant reductions in time to important postoperative recovery milestones, including postoperative days to first liquid diet (2.34 days vs. 3.68 days, *p* < 0.0001), to first solid food (3.45 days vs. 5.26 days, *p* < 0.0001), to first flatus (2.68 vs. 3.17, *p* < 0.0001) and to first stool (4.23 days vs. 5.53 days, *p* < 0.0001), in the integrated ERAS protocol compared with traditional group. Several studies evaluated elements of the ERAS care pathways in radical cystectomy and found benefits in early return to bowel function. Roth et al.^48^ in a prospective randomised trial on the role of parenteral nutrition in postoperative recovery from radical cystectomy concluded that postoperative parental nutrition is associated with a higher incidence of complications, mainly infections, and higher costs following radical cystectomy versus oral nutrition alone. Similar findings were reported by studies by Jensen et al.^49^

The postoperative complications following radical cystectomy were graded as per the Calvin Dindo classification system. One of the major postoperative complications following radical cystectomy was postoperative ileus in both cohorts. This is in accordance with the study by Tinoco and Lima^50^ in which the author reviewed the complications in the early postoperative period following radical cystectomy with urinary diversion and found that ileus was the most common complication reported in the literature. There was a statistically significant difference in postoperative ileus rate (31.91% vs. 55.32%, *p* value = 0.021) following radical cystectomy in the enhanced recovery cohort. This significant decrease is because Enhanced recovery programmes modify several factors that can contribute to ileus including minimising preoperative fasting and eliminating bowel preparation, opioid‐free analgesia, avoidance of nasogastric tube use and post‐surgery use of gut stimulating agents like chewing gum and early enteral feeding. Kouba et al.^51^ conducted a study on 102 patients who underwent radical cystectomy and urinary diversion for clinically localised bladder cancer. These patients were given chewing gum to begin on postoperative day 1, and compared with a group of 51 patients who underwent the same operation, this group served as a comparison (control) group in which no gum was dispensed. The time to flatus was shorter, the time to bowel movement was reduced and the length of hospital stay was shorter in patients who received gum compared with controls. Similar results on the positive effect of chewing gum on gut motility were noted by Choi, Kang et al.,^52^ Ziouziou et al.^53^ and Atkins et al.^54^

In our study, oral mechanical bowel preparation was omitted in the preoperative period in the ERAS cohort. Raynor et al.^55^ in a study eliminated preoperative mechanical bowel preparation in patients undergoing radical cystectomy and concluded that preoperative mechanical bowel preparation prior to radical cystectomy with urinary diversion does not demonstrate any significant advantage in perioperative outcomes, including gastrointestinal complications.

We avoided the routine use of nasogastric tubes in our study. Adamakis et al.^56^ in a randomised, prospective study to assess early NGT removal after radical cystectomy advocated early removal, independent of the selected type of urinary diversion, since it is not correlated with ileus and is advantageous in terms of patient comfort and earlier ambulation.

The need for parental nutrition was significantly reduced in the ERAS cohort compared with the traditional cohort (36.3% vs. 55.32, *p* value = 0.023). This decrease in the need for parental nutrition is attributed to the early use of oral nutrition and the consequent prevention of postoperative ileus. Declercq et al.^57^ studied the effect on parenteral nutrition of an improved recovery oral nutrition protocol after radical uncomplicated cystectomy and concluded that the implementation of enteral nutrition early in the postoperative period led to a reduction in the need for parental nutrition and improved recovery after uncomplicated cystectomy.

Regarding the other postoperative complication, there was a statistically insignificant reduction in postoperative fever (17.02% vs. 21.28, *p* value = 0.60), surgical site infections (10.64 vs. 14.9, *p* value = 0.536), intraabdominal collection (6.38% vs. 4.26, *p* value = 0.645), SSI requiring drainage (4.26 vs. 2.13, *p* value = 0.557), respiratory failure requiring ventilatory support (10.64 vs. 14.9, *p* value = 0.536) and multiorgan failure (4.26% vs. 6.38%, *p* value = 0.645). These results are similar to that reported by Pang et al.^58^ reporting the benefits of postoperative recovery without any increase in the complication rate.

In our study, the overall 30‐day mortality from radical cystectomy with ileal conduit was 7.45%, and the mortality rate of ERAS cohort compared with traditional cohort showed no statistical significance (6.38% vs. 8.51%, *p* value = 1). Similar results were obtained in studies by Casans‐Francés et al.^59^ showing no significant increase in mortality in ERAS as compared with the traditional group.

## 5 CONCLUSION

In conclusion, the implementation of the integrated ERAS approach has shown promising results in improving postoperative recovery and patient outcomes. The integrated ERAS approach facilitates a more efficient recovery process, potentially reducing healthcare costs and enhancing patient comfort.

## AUTHOR CONTRIBUTIONS

Each author contributed significantly to the conception, design, analysis and interpretation of data presented in this case report. (1) Dr WA—data collection and follow‐up of patients. (2) Dr SAM—design. (3) Prof AH—supervision. (4) Prof MSW—supervision. (5) Dr ARK—review of literature. (6) Dr SAP—discussion.

## CONFLICT OF INTEREST STATEMENT

The authors declare no competing interests that could potentially influence the objectivity or interpretation of the reported findings.

## Data Availability

Data and materials relevant to this case report are available upon request from the corresponding author for purposes of further scientific investigation and verification.

## References

[1] Kehlet H . Multimodal approach to control postoperative pathophysiology and rehabilitation. Br J Anaesth. 1997 May;78(5):606–617. 10.1093/bja/78.5.606 9175983

[2] Kehlet H . The surgical stress response: should it be prevented? Can. J. Surg. 1991;34(6):565–567. 1747833

[3] Bardram L , Funch‐Jensen P , Jensen P , et al. Recovery after laparoscopic colonic surgery with epidural analgesia, and early oral nutrition and mobilization. The Lancet. 1995;345(8952):763–764. 10.1016/S0140-6736(95)90643-6 7891489

[4] Kehlet H , Mogensen T . Hospital stay of 2 days after open sigmoidectomy with a multimodal rehabilitation programme. Br J Surg. 1999;86(2):227–230. 10100792 10.1046/j.1365-2168.1999.01023.x

[5] Cusack B , Buggy DJ . Anaesthesia, analgesia, and the surgical stress response. BJA Educ. 2020 Sep;20(9):321–328. 10.1016/j.bjae.2020.04.006 33456967 PMC7807970

[6] Ljungqvist O . Enhanced recovery after surgery and the ERAS® society. J Pancreatol. 2019;2(3):65–68. 10.1097/JP9.0000000000000025

[7] Ljungqvist O , Young‐Fadok T , Demartines N . The history of enhanced recovery after surgery and the ERAS society. J Laparoendosc Adv Surg Tech. 2017;27(9):860–862. 10.1089/lap.2017.0350 28795858

[8] Fearon KCH , Ljungqvist O , Meyenfeldt V , et al. Enhanced recovery after surgery: A consensus review of clinical care for patients undergoing colonic resection. Clin Nutr. 2005;24(3):466–477. 10.1016/j.clnu.2005.02.002 15896435

[9] Miller TE , Thacker JK , White WD , Mantyh C , Migaly J , Jin J , et al. Reduced length of hospital stay in colorectal surgery after implementation of an enhanced recovery protocol. Anesth Analg. 2014;118(5):1052–1061. 10.1213/ANE.0000000000000206 24781574

[10] Wind J , Polle S , Fung Kon Jin P , Dejong CH , von Meyenfeldt M , Ubbink DT , et al. Systematic review of enhanced recovery programmes in colonic surgery. Br J Surg. 2006;93(7):800–809. 10.1002/bjs.5384 16775831

[11] Khoo CK , Vickery CJ , Forsyth N , et al. A prospective randomized controlled trial of multimodal perioperative management protocol in patients undergoing elective colorectal resection for cancer. Ann Surg. 2007;245(6):867–872. 10.1097/01.sla.0000259219.08209.36 17522511 PMC1876970

[12] Baack Kukreja JE , Kiernan M , Schempp B , et al. Quality improvement in cystectomy care with enhanced recovery (QUICCER) study. BJU Int. 2017;119(1):38–49. 10.1111/bju.13521 27128851

[13] Yafi FA , Steinberg JR , Kassouf W . Contemporary management of muscle‐invasive bladder cancer. Int J Clin Oncol. 2008;13(6):504–509. 10.1007/s10147-008-0788-9 19093177

[14] Morii Y , Osawa T , Suzuki T , Shinohara N , Harabayashi T , Ishikawa T , et al. Cost comparison between open radical cystectomy, laparoscopic radical cystectomy, and robot‐assisted radical cystectomy for patients with bladder cancer: a systematic review of segmental costs. BMC Urol. 2019;19(1):110. 10.1186/s12894-019-0533-x 31703573 PMC6842244

[15] Krajewski W , Zdrojowy R , Tupikowski K , et al. How to lower postoperative complications after radical cystectomy—a review. Cent Eur J Urol. 2016;69(4):370–376. 10.5173/ceju.2016.880 PMC5260457 28127453

[16] Gurushankari B , Bilgi K , et al. Emerging concepts in enhanced recovery after surgery: potential functional adaptations to existing principles. Int J Adv Med Health Res. 2020;7(2):50–60.

[17] Kehlet H . Enhanced recovery after surgery (ERAS): good for now, but what about the future? Can J Anaesth. 2015;62(2):99–104. Journal canadien d'anesthesie. 10.1007/s12630-014-0261-3 25391731

[18] ERAS . 2022. ERAS Guidelines for Perioperative Care. https://erassociety.org/guidelines/

[19] Kehlet H , Wilmore DW . Evidence‐based surgical care and the evolution of fast‐track surgery. Ann Surg. 2008;248(2):189–198. 10.1097/SLA.0b013e31817f2c1a 18650627

[20] Hjort JD , Rud K , Kehlet H , et al. Standardising fast‐track surgical nursing care in Denmark. Br J Nurs. 2014;23(9):471–476. 10.12968/bjon.2014.23.9.471 24820811

[21] Watson DJ . Nurse coordinators and ERAS programs. Nurs Manage. 2018;49(1):42–49. 10.1097/01.NUMA.0000527718.90264.89 29287049

[22] Williams SB , Cumberbatch MG , Kamat AM , et al. Reporting radical cystectomy outcomes following implementation of enhanced recovery after surgery protocols: a systematic review and individual patient data meta‐analysis. Euro Urol. 2020;78(5):719–730. 10.1016/j.eururo.2020.06.039 32624275

[23] Tyson MD , Chang SS . Enhanced recovery pathways versus standard care after cystectomy: a meta‐analysis of the effect on perioperative outcomes. Eur Urol. 2016;70(6):995–1003. 10.1016/j.eururo.2016.05.031 27297680 PMC5149115

[24] Smith A , Anders M , Auffenberg G , et al. Optimizing outcomes in urologic surgery: postoperative. Am Urol Assoc. 2018.

[25] Lee G , Patel HV , Srivastava A , Ghodoussipour S . Updates on enhanced recovery after surgery for radical cystectomy. Ther Adv Urol. 2022 Jul;12(14) 17562872221109022. 10.1177/17562872221109022 PMC9280843 35844831

[26] Nayak B , Garg H , Goel R , et al. Contemporary outcomes of open radical cystectomy: a 5‐year experience from a tertiary care center. Indian J Surg Oncol. 2021 Mar;12(1):86–93. 10.1007/s13193-020-01226-z 33814837 PMC7960850

[27] Gupta, P. , Jain, M. , Kapoor, R. , et al. Impact of age and gender on the clinicopathological characteristics of bladder cancer. Indian J Urol. 2009;25(2):207–210. 19672348 10.4103/0970-1591.52916 PMC2710066

[28] Siegel RL , Miller KD , Fuchs HE , Jemal A . Cancer statistics, 2021. CA Cancer J Clin. 2021;71(1):7–33. 10.3322/caac.21654 33433946

[29] Hendri AZ , Khalilullah SA , Aditya GA . A preliminary outcome of modified enhanced recovery protocol versus standard of care in radical cystectomy: an Indonesian experience. Afr J Urol. 2021;27(1):111. 10.1186/s12301-021-00213-2

[30] Antoni S , Ferlay J , Soerjomataram I , Znaor A , Jemal A , Bray F . Bladder cancer incidence and mortality: a globaloverview and recent trends. Eur Urol. 2017;71(1):96–108. 10.1016/j.eururo.2016.06.010 27370177

[31] Hansel DE , Amin MB , Comperat E , Cote RJ , Knüchel R , Montironi R , et al. A contemporary update on pathology standards forbladder cancer: transurethral resection and radical cystectomy specimens. Eur Urol. 2013;63(2):321–332. 10.1016/j.eururo.2012.10.008 23088996

[32] Chalasani V , Chin JL , Izawa JI . Histologic variants of urothelial bladder cancer and nonurothelial histology in bladder cancer. Can Urol Assoc J. 2009;3(6 Suppl 4):S193–S198. 10.5489/cuaj.1195 20019984 PMC2792446

[33] Klaile Y , Schlack K , Boegemann M et.al. Variant histology in bladder cancer: how it should change the management in non‐muscle invasive and muscle invasive disease? Transl Androl Urol 2016 Oct;5(5):692–701, 10.21037/tau.2016.06.13 27785426 PMC5071184

[34] Piccirillo JF , Tierney RM , Costas I , et.al., Prognostic importance of comorbidity in a hospital‐based cancer registry. Jama 2004;291:2441–2447, 20, 10.1001/jama.291.20.2441 15161894

[35] Williams SB , Huo J , Chamie K , Hu JC , Giordano SH , Hoffman KE , et al. Underutilization of radical cystectomy among patients diagnosed with clinical stage T2 muscle‐invasive bladder cancer. Eur Urol Focus. 2017;3(2‐3):258–264. 10.1016/j.euf.2016.04.008 28753760

[36] Eisenberg MS , Boorjian SA , Cheville JC , Thompson RH , Thapa P , Kaushik D , et al. The SPARC score: a multifactorial outcome prediction model for patients undergoing radical cystectomy for bladder cancer. J Urol. 2013;190(6):2005–2010. 10.1016/j.juro.2013.06.022 23770147

[37] Noon AP , Albertsen PC , Thomas F , et al. Competing mortality in patients diagnosed with bladder cancer: evidence of undertreatment in the elderly and female patients. Br J Cancer. 2013;108(7):1534–1540. 10.1038/bjc.2013.106 23481180 PMC3629420

[38] Lee RK , Abol‐Enein H , Artibani W , et al. Urinary diversion after radical cystectomy for bladder cancer: options, patient selection, and outcomes. BJU Int. 2014;113(1):11–23. 10.1111/bju.12121 24330062

[39] Hanna P , Zabell J , Osman Y , Hussein MM , Mostafa M , Weight C , et al. Enhanced recovery after surgery (ERAS) following radical cystectomy: is it worth implementing for all patients? World J Urol. 2021;39(6):1927–1933. 10.1007/s00345-020-03435-1 32918095

[40] Moschini M , Stabile A , Mattei A , et al. Enhanced recovery after surgery (ERAS) in radical cystectomy patients: from consensus to evidences. Int Braz J Urol 2019 Jul‐Aug;45(4):655–657. 10.1590/s1677-5538.ibju.2019.04.02 31397986 PMC6837621

[41] Özdemir AT , Altinova S , et al. Is placement of pelvic drain indispensable after radical cystectomy, extended lymph node dissection, and orthotopic neobladder substitution? Turkish Journal of Medical Sciences. 2013;43(2). PMID: Article 14.

[42] Sung LH , Yuk HD . Enhanced recovery after surgery of patients undergoing radical cystectomy for bladder cancer. Transl Androl Urol. 2020 Dec;9(6):2986–2996. 10.21037/tau.2020.03.44 33457271 PMC7807364

[43] Beano H , He J , Hensel C , Worrilow W , et al. Safety of decreasing ureteral stent duration following radical cystectomy. World J Urol. 2021;39(2):473–479. 32303901 10.1007/s00345-020-03191-2

[44] Donat SM , Tan KS , Jibara G , et al. Intraoperative ureteral stent use at radical cystectomy is associated with higher 30‐day complication rates. J Urol. 2021 Feb;205(2):483–490. 10.1097/JU.0000000000001329 33238829 PMC8162033

[45] Semerjian A , Milbar N , Kates M , et al. Hospital charges and length of stay following radical cystectomy in the enhanced recovery after surgery era. Urology. 2018;111:86–91. 10.1016/j.urology.2017.09.010 29032237

[46] Pramod SV , Safriadi F , Hernowo BS , et al. Modified enhanced recovery after surgery protocol versus nonenhanced recovery after surgery in radical cystectomy surgery (preliminary study). Urol Sci. 2020;31(4):177–182. 10.4103/UROS.UROS_8_20

[47] Wessels F , Lenhart M , Kowalewski KF , Braun V , Terboven T , Roghmann F , et al. Early recovery after surgery for radical cystectomy: comprehensive assessment and meta‐analysis of existing protocols. World J Urol. 2020;38(12):3139–3153. 10.1007/s00345-020-03133-y 32124020 PMC7716903

[48] Roth B , Birkhäuser FD , Zehnder P , et al. Parenteral nutrition does not improve postoperative recovery from radical cystectomy: results of a prospective randomised trial. Eur Urol. 2013;63(3):475–482. 10.1016/j.eururo.2012.05.052 22695241

[49] Jensen BT , Petersen AK , Jensen JB , et al. Efficacy of a multiprofessional rehabilitation programme in radical cystectomy pathways: a prospective randomized controlled trial. Scand J Urol. 2015;49(2):133–141. 10.3109/21681805.2014.967810 25331367

[50] Tinoco CL , Lima E . Urinary diversions for radical cystectomy: a review of complications and their management. Mini‐Invasive Surg. 2021;5:28.

[51] Kouba EJ , Wallen EM , Pruthi RS . Gum chewing stimulates bowel motility in patients undergoing radical cystectomy with urinary diversion. Urology. 2007;70(6):1053–1056. 10.1016/j.urology.2007.07.048 18158012

[52] Choi H , Kang SH , Yoon DK , Kang , et al. Chewing gum has a stimulatory effect on bowel motility in patients after open or robotic radical cystectomy for bladder cancer: a prospective randomized comparative study. Urology. 2011;77(4):884–890. 10.1016/j.urology.2010.06.042 20950844

[53] Ziouziou I , Ammani A , Karmouni T , et al. Le chewing‐gum améliore‐t‐il les résultats postopératoires chez les patients opérés d'une cystectomie radicale ? Revue systématique de la littérature et méta‐analyse [Does chewing gum improve postoperative results in patients undergoing radical cystectomy? A systematic review of literature and meta‐analysis]. Prog Urol. 2017;27(10):513–520. 10.1016/j.purol.2017.06.005 28734774

[54] Atkins CS , Tubog TD , Schaffer SK . Chewing gum after radical cystectomy with urinary diversion for recovery of intestinal function: A systematic review and meta‐analysis. J Perianesth Nursing: Official Journal of the American Society of PeriAnesthesia Nurses. 2022;37(4):467–473. 10.1016/j.jopan.2021.10.003 35272926

[55] Raynor MC , Lavien G , Nielsen M , Wallen , et al. Elimination of preoperative mechanical bowel preparation in patients undergoing cystectomy and urinary diversion. Urol Oncol. 2013;31(1):32–35. 10.1016/j.urolonc.2010.11.002 21719323

[56] Adamakis I , Tyritzis SI , Koutalellis G , et al. Early removal of nasogastric tube is beneficial for patients undergoing radical cystectomy with urinary diversion. Int Braz J Urol. 2011;37(1):42–48. 10.1590/S1677-55382011000100006 21385479

[57] Declercq P , de Win G , van der Aa F , et al. Effect on parenteral nutrition of an improved recovery oral nutrition protocol after radical uncomplicated cystectomy. European Journal of Hospital Pharmacy: Science and Practice. 2012;19(2):219–21219. 10.1136/ejhpharm-2012-000074.351

[58] Pang KH , Groves R , Venugopal S , et al. Prospective implementation of enhanced recovery after surgery protocols to radical cystectomy. Eur Urol. 2018;73(3):363–371. 10.1016/j.eururo.2017.07.031 28801130

[59] Casans‐Francés, R. , Roberto‐Alcácer, A. , García‐Lecina, A. , et al. Impact of an enhanced recovery after surgery programme in radical cystectomy. A cohort‐comparative study. Rev Esp Anestesiol Reanim (English Edition) 2017;64(6):313–322. 10.1016/j.redar.2016.12.002 28214097

